# Global, regional, and national burdens of traumatic brain injury from 1990 to 2021

**DOI:** 10.3389/fpubh.2025.1556147

**Published:** 2025-04-14

**Authors:** Junqing Yan, Chao Wang, Bangqing Sun

**Affiliations:** Nanxiang Branch of Ruijin Hospital, Shanghai, China

**Keywords:** traumatic brain injury, global health, burden of disease, public health, epidemiology

## Abstract

**Background:**

Of all the injuries on a global scale, traumatic brain injury (TBI) has the most serious consequences for the individual. Depending on its severity, it can be classified as minor, moderate, or severe, but even minor TBI can sometimes still cause severe functional deficits. This study seeks to assess the latest burden of TBI and analyze their differences in terms of country, age, sex, and cause.

**Methods:**

Based on the Global Burden of Diseases database, the incidence, years lived with disability (YLDs), and causes of total head injuries, minor TBI, and moderate/severe TBI from 1990 to 2021 were analyzed separately by sex, age group, and region.

**Results:**

In 2021, there were 20,837,465 [95% uncertainty interval (UI): 18,128,306–23,839,393] new cases of TBI worldwide, with an age-standardized incidence of 259 cases per 100,000 population (95% UI: 226–296). From 1990 to 2021, there was a decline in global age-standardized incidence (estimated annual percentage change: −0.11, 95% UI: −0.18% to −0.04%). In 2021, countries with higher rates will be mainly in Central and Eastern Europe and the Middle East. In 2021, the global incidence of TBI in all age groups was higher in men than in women. Falls are the leading cause for most age groups in most areas.

**Discussion:**

TBI still accounts for a significant portion of the global injury burden in 2021, but differences do exist between countries. This study introduced the possibility of TBI with different degrees and the trend of injury causes in different age groups and regions from 1990 to 2021, providing a basis for further research on injury causes in different regions and formulating corresponding policies and protection measures in the future.

## 1 Introduction

Traumatic Brain Injury (TBI) is a major public health issue with far-reaching consequences both for individual health and for society. The burden of TBI is evident not only in terms of mortality rates but also through the long-term disabilities that survivors experience. Some TBI patients face death early in the course of hospitalization, with elevated mortality persisting for up years after the injury (1–3). For those who survive, TBI can result in prolonged physical, neurological, and psychological impairments, with about half of the affected individuals experiencing moderate to severe long-term disabilities (4).

The social costs of TBI are also enormous. Many TBI incidents occur in younger individuals, often leading to lifelong care requirements and specialized accommodations (5). One study showed that in the acute phase, the average additional cost of TBI is $22,000, which continues to accumulate over a period of up to 6 years, leading to long-term medical, rehabilitation, and support needs (6). The total costs associated with TBI are comparable to the total costs of addressing cardiovascular diseases, cancer, and diabetes (7). Furthermore, injuries that occur during productive years often result in lifelong care requirements and specialized accommodations, as well as a loss of productivity, imposing a significant economic burden on both the individual and society (8).

Addressing TBI requires a comprehensive understanding of its epidemiology, etiology, and the identification of at-risk populations. Standardized and up-to-date epidemiological data are essential for the development of effective prevention and treatment strategies. The Global Burden of Disease (GBD) 2021 study, with its improved data sources, replaces the previous GBD 2019 results, making it crucial to update our understanding of the burden of TBI (9, 10).

This study uses data from GBD 2021 to provide a comprehensive overview of the incidence, years lived with disability (YLDs), and trends in the mechanisms of different level of TBI across global and regional populations. It also explores variations in TBI impact by age and gender from 1990 to 2021, providing updated insights that are critical for shaping future prevention efforts.

## 2 Materials and methods

### 2.1 Overview

The method used to estimate the burden of TBI is based on the framework of the GBD 2021 study. The GBD 2021 dataset provides global burden data for 371 diseases and injuries, categorized by gender, age, and cause of morbidity across 204 regions and countries, from 1990 to 2021 (10). This global health estimation follows the guidelines of the Global Health Estimates Reporting (GATHER) statement and the Strengthening the Reporting of Observational Studies in Epidemiology (STROBE) statement (11, 12).

This study extracted number and age-standardized rates (ASR) data from GBD 2021 on TBI incidence, prevalence, and YLDs, and performed secondary analyses on these datasets.

### 2.2 Case definition

In this study, TBI was defined according to the International Classification of Diseases, 10th edition (ICD-10). The ICD-10 codes for TBI are F07.2, F07.8, F07.81, F07.89, F07.9, S06, S07, T90.2, and T90.5. Minor TBI is specifically defined as S06.1, while all other codes are categorized as moderate/severe TBI (9). Furthermore, TBI is considered as an injury type rather than an injury cause. TBI was not classified as a cause of death in the GBD 2021 study, therefore, mortality and years of life lost (YLL) are not included in this analysis.

### 2.3 Data sources

The GBD database is publicly available on the Institute for Health Metrics and Evaluation (IHME) website.^1^ The data is derived from hospital and emergency department records of patients requiring medical care. Bayesian meta-regression tools (DisMod-MR 2.1) were used to model the incidence of each injury cause. In cases involving multiple types of injuries, the highest-ranked injury according to the GBD severity hierarchy was used for matching (10). YLDs were calculated by multiplying the prevalence by disability weights, which were derived from population and internet surveys evaluating disability conditions (13).

Due to sampling errors and non-sampling variance, the same methods were followed as described in the GBD 2021 study to calculate 95% uncertainty intervals (UI). Typically, the UI is derived from 1000 parameter estimates at each drawing level. The 95% UI is defined as the 25th and 975th values from the ordered set of 1,000 estimates (10).

### 2.4 Statistical analysis

The present study analyzed the global and regional TBI incidence, prevalence, and YLDs in 2021 to assess the overall burden of TBI. ASRs of incidence, prevalence, and YLDs were further compared across gender, age, and injury cause. To quantify the dynamics of each indicator from 1990 to 2021, the estimated annual percentage change (EAPC) in ASR was calculated using a regression model.

Y=α+βX+ϵ, where Y is the natural logarithm of ASR, and X is the calendar year. Using the regression model and ASR data from recent years across regions, the coefficients *α* and *β* were estimated via least squares. The EAPC was calculated as: $\mathrm{EAPC}=100\times \left(\exp\left(\beta \right)-1\right)$ .

The corresponding 95% confidence intervals for the coefficient were also calculated. An EAPC > 0 indicates an increasing ASR, while an EAPC < 0 indicates a decreasing ASR (14).

Additionally, we conducted a population-based analysis to explore the distribution of TBI across different population groups, including age, gender, and specific subgroups. Data for males and females were stratified by age group. All statistical analyses were performed using R software, and results were visualized using the ggplot2 package.

## 3 Result

### 3.1 Global and regional perspectives

In 2021, there were 20,837,466 cases of head injuries worldwide (95% UI: 18,128,307–23,839,394), with 56.63% classified as moderate/severe TBI. Regionally, East Asia had the highest number of cases, 4,313,633 (95% UI: 3,671,116–4,998,872), consistent with data from 1990. Of these, 69% were moderate/severe TBI.

After age-standardization, the global age-standardized incidence rate for 2021 was 259 cases per 100,000 people (95% UI: 226–296). Regionally, the highest age-standardized incidence rates were observed in Eastern Europe (522, 95% UI: 454–602), Australasia (479, 95% UI: 355–652), and Central Europe (479, 95% UI: 405–561), all far exceeding other regions (where the rates were below 400). In contrast, regions with the lowest age-standardized incidence rates included Central Sub-Saharan Africa (183, 95% UI: 163–205), Eastern Sub-Saharan Africa (167, 95% UI: 148–191), and Western Sub-Saharan Africa (162, 95% UI: 144–181), with all regions exhibiting a higher incidence in males compared to females.

In terms of severity, the proportion of moderate/severe TBI cases has increased from 1990 to 2021. With the exception of high-income North America (29.23%), Western Europe (27.94%), High-income Asia Pacific (27.29%), Australasia (20.86%), and Southern Latin America (15.89%), the majority of regions report moderate/severe TBI constituting more than 50% of cases.

A dynamic trend analysis from 1990 to 2021 shows a slight global decline in the age-standardized incidence rate (EAPC: −0.8, 95% UI: −0.85 to −0.75). Most regions exhibited a decreasing trend, except for North Africa and the Middle East (0, 95% UI: −0.17 to 0.16), the Caribbean (0.46, 95% UI: −0.65 to 1.59), and Oceania (0.13, 95% UI: −0.39 to 0.65).

In 2021, the prevalence number of TBI was 37,928,494 (95% UI: 36,333,776–39,771,326), with East Asia (9,523,998, 95% UI: 9,077,425–10,041,469) remaining the region with the highest number of cases, consistent with data from 1990.

Globally, the age-standardized prevalence rate in 2021 was 448 cases per 100,000 people (95% UI: 429–470). The highest age-standardized prevalence rates were observed in Eastern Europe (276, 95% UI: 253–312), Central Europe (771, 95% UI: 737–812), and Tropical Latin America (643, 95% UI: 616–676), far surpassing other regions. In contrast, Eastern Sub-Saharan Africa (276, 95% UI: 253–312) and Western Sub-Saharan Africa (256, 95% UI: 245–269) had the lowest age-standardized prevalence rates.

Age-standardized prevalence rates from 1990 to 2021 show a global decline (EAPC: −0.68, 95% UI: −0.72 to −0.64). Only the Caribbean (0.71, 95% UI: 0.51 to 0.91) and Oceania (0.57, 95% UI: 0.52 to 0.62) showed an increasing trend. Other regions experienced declines, with the largest reductions seen in Western Europe (−1.12, 95% UI: −1.18 to −1.06), Eastern Europe (−1.15, 95% UI: −1.37 to −0.92), and Southern Sub-Saharan Africa (−1.73, 95% UI: −1.82 to −1.65), all exceeding a 1% decrease.

In terms of Years Lived with Disability (YLD), East Asia (1,409,201, 95% UI: 987,681–1,922,142) and South Asia (920,914, 95% UI: 652,975–1,243,160) contributed significantly higher YLDs than other regions. After age-standardization, Eastern Europe (130, 95% UI: 91–178) and Central Europe (113, 95% UI: 79–154) had the highest age-standardized YLDs, both exceeding 100. Interestingly, these regions did not have the highest proportions of severe TBI (Table 1).

**Table 1:** Incidence, prevalence, moderate to severe TBI ratio and YLDs of TBI and age-standardized rates in 2021 and estimated annual percent-change in age-standardized rates by GBD region from 1990 to 2021.

Location	Incidence				Prevalence				YLDs		
	2021 counts	2021 age-standardized rate (per 100,000)	2021 moderate to severe TBI ratio	Estimated annual percentage change from 1990 to 2021, % (95% UI)	2021 counts	2021 age-standardized rate (per 100,000)	2021 moderate to severe TBI ratio	Estimated annual percentage change from 1990 to 2021, % (95% UI)	2021 counts	2021 age-standardized rate (per 100,000)	Estimated annual percentage change from 1990 to 2021, % (95% UI)
Global											
Both	20,837,466 (18,128,307–23,839,394)	259 (226–296)	56.63%	−0.8 (−0.85 to −0.75)	37,928,494 (36,333,776–39,771,326)	448 (429–470)	76.59%	−0.68 (−0.72 to −0.64)	5,480,354 (3,870,216–7,331,092)	65 (46–87)	−0.66 (−0.7 to −0.62)
Male	13,856,451 (12,201,551–15,600,382)	347 (306–390)	57.38%	−0.8 (−0.84 to −0.76)	25,497,575 (24,435,087–26,685,824)	616 (591–645)	78.32%	−0.66 (−0.7 to −0.62)	3,727,418 (2,630,852–4,998,674)	90 (63–121)	−0.63 (−0.68 to −0.59)
Female	6,981,015 (5,749,070–8,314,257)	170 (141–202)	55.13%	−0.77 (−0.86 to −0.69)	12,430,919 (11,828,468–13,122,817)	284 (270–299)	73.05%	−0.7 (−0.74 to −0.65)	1,752,937 (1,247,139–2,346,109)	40 (28–54)	−0.69 (−0.74 to −0.65)
Andean Latin America											
Both	152,864 (133,550–173,685)	230 (201–260)	55.14%	−0.42 (−0.51 to −0.33)	263,002 (251,379–274,740)	410 (392–428)	81.73%	−0.17 (−0.27 to −0.07)	38,823 (27,406–52,570)	60 (43–82)	−0.18 (−0.28 to −0.08)
Male	112,424 (98,751–126,403)	335 (294–377)	55.90%	−0.47 (−0.58 to −0.37)	194,204 (185,044–202,843)	618 (589–645)	82.56%	−0.16 (−0.27 to −0.06)	28,846 (20,360–38,966)	91 (65–124)	−0.17 (−0.28 to −0.07)
Female	40,439 (34,228–47,363)	124 (105–146)	53.04%	−0.33 (−0.4 to −0.25)	68,798 (65,785–72,434)	211 (202–222)	79.39%	−0.18 (−0.28 to −0.08)	9,977 (7,056–13,395)	31 (22–41)	−0.2 (−0.29 to −0.1)
Australasia											
Both	139,248 (105,001–182,132)	479 (355–652)	20.86%	−0.58 (−0.65 to −0.51)	210,178 (198,130–223,338)	518 (483–557)	35.44%	−0.66 (−0.73 to −0.6)	27,273 (19,167–36,326)	68 (48–91)	−0.67 (−0.73 to −0.61)
Male	84,713 (64,922–110,681)	587 (445–778)	20.86%	−0.76 (−0.84 to −0.68)	126,572 (119,022–134,599)	651 (610–695)	37.24%	−0.8 (−0.87 to −0.72)	16,608 (11,597–22,255)	86 (60–115)	−0.79 (−0.87 to −0.72)
Female	54,534 (39,885–74,218)	370 (263–536)	20.85%	−0.27 (−0.33 to −0.2)	83,606 (78,041–89,984)	390 (361–426)	32.71%	−0.41 (−0.47 to −0.36)	10,665 (7,526–14,163)	50 (35–67)	−0.44 (−0.49 to −0.38)
Caribbean											
Both	144,663 (125,192–163,811)	299 (260–339)	61.06%	0.46 (−0.65 to 1.59)	262,836 (243,264–290,669)	514 (473–573)	83.53%	0.71 (0.51 to 0.91)	38,527 (27,780–49,939)	76 (54–98)	0.7 (0.47 to 0.93)
Male	94,852 (84,154–105,527)	400 (356–447)	59.72%	0.31 (−0.53 to 1.15)	170,936 (162,763–180,830)	690 (656–732)	83.49%	0.35 (0.24 to 0.46)	25,288 (17,986–33,423)	102 (73–135)	0.34 (0.21 to 0.47)
Female	49,811 (40,696–59,653)	198 (162–238)	63.61%	0.66 (−0.83 to 2.17)	91,900 (80,313–111,361)	347 (299–428)	83.60%	1.45 (1.08 to 1.83)	13,239 (9,733–17,200)	50 (37–65)	1.46 (1.03 to 1.88)
Central Asia											
Both	269,848 (237,410–305,727)	280 (247–319)	52.34%	−1.05 (−1.23 to −0.87)	450,495 (430,153–470,411)	471 (450–491)	80.35%	−0.87 (−0.91 to −0.83)	66,723 (46,504–90,670)	69 (48–94)	−0.88 (−0.92 to −0.84)
Male	201,209 (177,550–226,878)	419 (371–473)	53.34%	−1.19 (−1.38 to −1)	329,301 (314,721–344,407)	716 (685–748)	81.11%	−1 (−1.05 to −0.96)	49,100 (34,114–66,935)	106 (74–144)	−1.01 (−1.06 to −0.96)
Female	68,639 (57,998–80,914)	144 (121–170)	49.43%	−0.73 (−0.88 to −0.57)	121,194 (116,101–127,201)	247 (237–259)	78.28%	−0.6 (−0.67 to −0.53)	17,623 (12,497–23,698)	36 (25–48)	−0.61 (−0.68 to −0.54)
Central Europe											
Both	601,947 (506,536–701,295)	479 (405–561)	63.57%	−1.22 (−1.28 to −1.15)	1,276,908 (1,217,810–1,343,114)	771 (737–812)	81.42%	−1 (−1.07 to −0.92)	184,635 (129,916–250,590)	113 (79–154)	−0.99 (−1.07 to −0.92)
Male	407,518 (349,517–465,719)	676 (578–784)	62.43%	−1.24 (−1.31 to −1.16)	868,586 (828,945–914,448)	1,118 (1,068–1,178)	82.00%	−1 (−1.08 to −0.93)	126,522 (88,678–172,195)	164 (114–224)	−1 (−1.08 to −0.92)
Female	194,429 (152,360–237,111)	281 (228–339)	65.96%	−1.14 (−1.21 to −1.08)	408,322 (385,166–432,049)	445 (421–469)	80.17%	−1 (−1.09 to −0.91)	58,112 (41,261–78,782)	64 (45–88)	−1 (−1.09 to −0.91)
Central Latin America											
Both	810,783 (714,280–915,908)	318 (280–361)	57.84%	−0.75 (−0.93 to −0.58)	1,489,308 (1,426,868–1,556,450)	570 (546–595)	82.69%	−0.65 (−0.8 to −0.5)	219,154 (153,910–296,100)	84 (59–113)	−0.65 (−0.8 to −0.5)
Male	595,488 (531,731–663,795)	474 (424–528)	59.60%	−0.75 (−0.91 to −0.59)	1,111,100 (1,064,838–1,159,544)	894 (857–933)	83.89%	−0.62 (−0.78 to −0.46)	164,640 (115,299–222,512)	132 (93–178)	−0.62 (−0.78 to −0.47)
Female	215,295 (177,605–256,608)	171 (140–204)	52.95%	−0.76 (−1.01 to −0.52)	378,208 (359,295–399,970)	279 (265–295)	79.15%	−0.59 (−0.75 to −0.43)	54,514 (38,479–73,414)	40 (28–54)	−0.6 (−0.76 to −0.45)
Central Sub-Saharan Africa											
Both	224,834 (200,748–253,866)	183 (163–205)	55.64%	−1.01 (−1.55 to −0.48)	290,898 (269,365–319,018)	319 (298–349)	82.27%	−0.01 (−0.1 to 0.07)	43,145 (30,984–56,807)	47 (33–61)	−0.01 (−0.09 to 0.08)
Male	151,235 (135,628–170,201)	246 (221–273)	57.57%	−1.06 (−1.69 to −0.42)	200,535 (183,522–223,194)	445 (411–491)	83.46%	0.16 (0.05 to 0.27)	29,933 (21,521–39,202)	65 (47–85)	0.16 (0.06 to 0.27)
Female	73,600 (63,633–85,577)	122 (106–141)	51.66%	−0.93 (−1.25 to −0.62)	90,363 (85,374–96,308)	200 (189–213)	79.63%	−0.41 (−0.46 to −0.36)	13,212 (9,179–17,701)	29 (20–38)	−0.41 (−0.46 to −0.35)
East Asia											
Both	4,313,633 (3,671,116–4,998,872)	263 (225–305)	69.85%	−0.16 (−0.35 to 0.03)	9,523,998 (9,077,425–10,041,469)	477 (456–501)	85.46%	−0.14 (−0.27 to −0.01)	1,409,201 (987,681–1,922,142)	71 (49–97)	−0.16 (−0.29 to −0.04)
Male	2,837,309 (2,435,824–3,243,290)	347 (299–398)	69.65%	−0.02 (−0.19 to 0.15)	6,343,450 (6,046,568–6,687,939)	637 (608–670)	86.07%	0 (−0.11 to 0.12)	946,268 (663,162–1,295,434)	95 (66–130)	−0.02 (−0.13 to 0.1)
Female	1,476,324 (1,200,760–1,780,577)	175 (145–210)	70.24%	−0.38 (−0.62 to −0.15)	3,180,547 (3,023,845–3,349,327)	314 (299–330)	84.24%	−0.36 (−0.51 to −0.21)	462,933 (326,215–628,032)	46 (32–63)	−0.38 (−0.53 to −0.24)
Eastern Europe											
Both	1,115,069 (963,661–1,274,684)	522 (454–602)	60.61%	−1.32 (−1.6 to −1.04)	2,492,282 (2,372,412–2,618,739)	889 (847–934)	81.74%	−1.15 (−1.37 to −0.92)	361,906 (253,832–493,680)	130 (91–178)	−1.14 (−1.36 to −0.93)
Male	824,738 (718,713–939,383)	799 (700–914)	61.12%	−1.38 (−1.67 to −1.09)	1,745,350 (1,657,988–1,843,711)	1,396 (1,326–1,475)	82.37%	−1.17 (−1.37 to −0.96)	255,492 (179,078–349,726)	205 (144–280)	−1.16 (−1.36 to −0.96)
Female	290,330 (244,960–339,407)	261 (217–308)	59.14%	−1.11 (−1.38 to −0.83)	746,931 (709,582–785,204)	466 (443–489)	80.26%	−1.05 (−1.33 to −0.78)	106,413 (74,689–144,134)	67 (47–92)	−1.06 (−1.34 to −0.78)
Eastern Sub-Saharan Africa											
Both	620,976 (544,556–720,066)	167 (148–191)	51.69%	−1.48 (−1.96 to −0.99)	748,723 (682,271–849,678)	276 (253–312)	80.15%	−0.37 (−0.47 to −0.27)	110,855 (80,127–144,604)	40 (29–52)	−0.37 (−0.47 to −0.27)
Male	430,704 (378,682–501,605)	233 (208–264)	54.66%	−1.49 (−1.99 to −0.98)	537,530 (484,830–618,635)	403 (365–461)	82.13%	−0.26 (−0.38 to −0.15)	80,274 (58,138–103,922)	59 (43–76)	−0.27 (−0.39 to −0.16)
Female	190,273 (161,710–223,111)	105 (90–123)	44.98%	−1.42 (−1.89 to −0.95)	211,193 (196,247–233,387)	156 (145–173)	75.11%	−0.57 (−0.65 to −0.5)	30,581 (21,950–40,623)	22 (16–30)	−0.58 (−0.65 to −0.5)
High-income Asia Pacific											
Both	446,418 (357,113–551,239)	243 (191–313)	27.29%	−1.71 (−1.81 to −1.61)	922,120 (876,101–972,923)	303 (287–321)	39.61%	−1.67 (−1.77 to −1.57)	121,024 (85,487–160,286)	40 (28–54)	−1.67 (−1.77 to −1.56)
Male	284,059 (231,084–349,232)	309 (247–393)	28.08%	−1.88 (−1.98 to −1.77)	569,797 (540,463–599,396)	394 (375–417)	41.88%	−1.84 (−1.95 to −1.73)	75,582 (53,266–100,828)	53 (37–71)	−1.83 (−1.94 to −1.73)
Female	162,358 (124,925–206,179)	176 (133–238)	25.91%	−1.41 (−1.51 to −1.31)	352,323 (332,513–377,056)	214 (200–230)	35.94%	−1.44 (−1.54 to −1.33)	45,442 (32,184–60,047)	28 (20–38)	−1.44 (−1.54 to −1.33)
High-income North America											
Both	1,100,572 (895,390–1,336,998)	269 (223–324)	29.23%	−1.16 (−1.35 to −0.97)	2,052,302 (1,945,820–2,160,393)	384 (365–403)	41.98%	−0.93 (−1.07 to −0.79)	265,812 (187,933–353,325)	50 (35–67)	−0.97 (−1.11 to −0.83)
Male	652,884 (547,557–772,880)	335 (283–396)	29.70%	−1.31 (−1.48 to −1.15)	1,198,621 (1,143,028–1,256,412)	483 (461–507)	43.87%	−1.1 (−1.23 to −0.98)	157,208 (110,812–208,387)	64 (45–85)	−1.13 (−1.25 to −1.01)
Female	447,689 (350,559–564,730)	204 (164–253)	28.56%	−0.89 (−1.13 to −0.65)	853,681 (800,116–908,496)	290 (273–308)	39.31%	−0.69 (−0.86 to −0.52)	108,604 (76,815–145,345)	37 (26–50)	−0.73 (−0.89 to −0.56)
North Africa and Middle East											
Both	2,050,666 (1,801,676–2,347,332)	333 (293–380)	57.46%	0 (−0.17 to 0.16)	3,447,607 (3,204,256–3,744,044)	593 (553–642)	83.42%	−0.45 (−0.47 to −0.43)	509,844 (367,555–666,956)	87 (63–114)	−0.47 (−0.49 to −0.45)
Male	1,490,368 (1,312,583–1,697,385)	460 (406–522)	58.90%	0.12 (−0.06 to 0.31)	2,551,782 (2,361,118–2,800,825)	837 (776–916)	84.42%	−0.39 (−0.41 to −0.36)	380,263 (274,533–496,464)	124 (90–161)	−0.4 (−0.42 to −0.38)
Female	560,299 (479,568–655,368)	196 (168–229)	53.61%	−0.39 (−0.52 to −0.25)	895,825 (845,677–956,220)	329 (311–350)	80.57%	−0.69 (−0.74 to −0.64)	129,580 (92,608–169,775)	47 (34–62)	−0.73 (−0.77 to −0.68)
Oceania											
Both	28,899 (25,664–32,512)	229 (204–259)	59.41%	0.13 (−0.39 to 0.65)	43,104 (40,952–45,459)	401 (382–421)	84.07%	0.57 (0.52 to 0.62)	6,452 (4,603–8,834)	59 (42–81)	0.56 (0.51 to 0.62)
Male	17,838 (16,014–19,769)	257 (232–284)	63.05%	0.02 (−0.39 to 0.44)	27,519 (26,111–29,183)	479 (455–506)	86.07%	0.47 (0.43 to 0.52)	4,161 (2,912–5,776)	72 (50–100)	0.48 (0.42 to 0.53)
Female	11,061 (9,201–13,082)	198 (161–238)	53.54%	0.3 (−0.36 to 0.97)	15,584 (14,673–16,749)	317 (299–338)	80.54%	0.74 (0.64 to 0.84)	2,291 (1,621–3,074)	46 (33–62)	0.73 (0.62 to 0.84)
South Asia											
Both	4,149,217 (3,543,281–4,823,244)	243 (204–283)	62.13%	−0.5 (−0.64 to −0.36)	6,311,107 (5,994,081–6,657,380)	383 (363–406)	82.50%	−0.06 (−0.14 to 0.02)	920,914 (652,975–1,243,160)	55 (39–75)	−0.06 (−0.14 to 0.02)
Male	2,521,318 (2,217,836–2,867,806)	282 (247–321)	62.24%	−0.44 (−0.57 to −0.31)	3,977,889 (3,778,198–4,183,021)	471 (449–496)	83.74%	0 (−0.1 to 0.11)	588,922 (415,881–794,932)	69 (49–93)	0.02 (−0.09 to 0.12)
Female	1,627,898 (1,305,756–1,965,084)	201 (158–244)	61.97%	−0.58 (−0.78 to −0.38)	2,333,217 (2,179,348–2,501,045)	293 (272–315)	80.39%	−0.1 (−0.14 to −0.05)	331,992 (236,806–443,287)	41 (29–55)	−0.1 (−0.15 to −0.06)
Southeast Asia											
Both	1,387,482 (1,217,084–1,564,118)	201 (175–227)	61.36%	−0.86 (−1.2 to −0.52)	2,572,303 (2,438,853–2,741,690)	360 (342–383)	83.59%	−0.71 (−0.75 to −0.67)	381,660 (273,314–505,887)	53 (38–71)	−0.72 (−0.76 to −0.67)
Male	956,901 (855,044–1,060,762)	273 (244–303)	61.85%	−0.91 (−1.13 to −0.69)	1,797,916 (1,703,084–1,921,668)	509 (483–543)	84.45%	−0.78 (−0.81 to −0.74)	268,897 (193,360–358,639)	76 (54–101)	−0.79 (−0.82 to −0.75)
Female	430,581 (357,414–504,672)	128 (105–150)	60.26%	−0.84 (−1.4 to −0.27)	774,387 (725,382–833,442)	217 (203–233)	81.59%	−0.58 (−0.66 to −0.5)	112,763 (81,418–148,221)	31 (23–42)	−0.59 (−0.68 to −0.5)
Southern Latin America											
Both	238,178 (184,978–321,973)	363 (280–496)	15.89%	−0.11 (−0.26 to 0.03)	317,384 (300,858–337,514)	410 (386–438)	34.47%	−0.08 (−0.19 to 0.04)	41,628 (29,466–55,531)	54 (38–72)	−0.07 (−0.18 to 0.04)
Male	162,876 (128,204–217,233)	494 (387–662)	16.19%	−0.31 (−0.45 to −0.16)	212,213 (200,737–225,169)	578 (546–615)	36.22%	−0.2 (−0.32 to −0.09)	28,130 (19,681–37,738)	77 (54–103)	−0.19 (−0.31 to −0.08)
Female	75,301 (55,900–104,986)	232 (168–331)	15.26%	0.28 (0.12 to 0.45)	105,170 (98,522–113,611)	255 (238–277)	30.95%	0.18 (0.06 to 0.29)	13,498 (9,688–18,082)	33 (24–44)	0.17 (0.05 to 0.29)
Southern Sub-Saharan Africa											
Both	203,466 (176,836–229,679)	249 (218–280)	63.79%	−1.35 (−1.48 to −1.22)	324,082 (305,063–344,922)	435 (409–462)	84.14%	−1.73 (−1.82 to −1.65)	47,961 (33,098–65,222)	64 (44–86)	−1.76 (−1.85 to −1.67)
Male	157,019 (135,953–177,744)	391 (343–440)	65.38%	−1.26 (−1.4 to −1.11)	246,753 (230,470–263,851)	703 (658–749)	85.00%	−1.53 (−1.6 to −1.46)	36,812 (25,356–50,333)	104 (72–141)	−1.56 (−1.63 to −1.49)
Female	46,447 (40,178–53,282)	115 (99–131)	58.41%	−1.78 (−1.92 to −1.65)	77,329 (73,477–82,304)	200 (191–213)	81.37%	−2.32 (−2.42 to −2.21)	11,149 (7,646–14,984)	29 (20–39)	−2.36 (−2.47 to −2.25)
Tropical Latin America											
Both	827,052 (722,081–948,791)	351 (305–403)	61.28%	−0.63 (−0.69 to −0.57)	1,647,002 (1,576,924–1,732,312)	643 (616–676)	83.82%	−0.58 (−0.61 to −0.54)	240,869 (169,319–325,629)	94 (66–127)	−0.56 (−0.6 to −0.53)
Male	623,420 (548,254–705,841)	541 (476–612)	61.32%	−0.59 (−0.66 to −0.53)	1,258,026 (1,203,586–1,327,562)	1,033 (989–1,090)	84.49%	−0.5 (−0.54 to −0.47)	185,352 (129,718–249,800)	152 (106–205)	−0.49 (−0.52 to −0.46)
Female	203,632 (167,130–244,608)	167 (137–201)	61.16%	−0.81 (−0.88 to −0.74)	388,975 (368,687–409,075)	288 (273–303)	81.63%	−0.73 (−0.78 to −0.68)	55,518 (39,409–74,977)	41 (29–56)	−0.73 (−0.78 to −0.69)
Western Europe											
Both	1,323,853 (1,043,018–1,667,371)	300 (234–381)	27.94%	−1.04 (−1.1 to −0.98)	2,491,552 (2,352,567–2,640,652)	376 (354–400)	40.01%	−1.12 (−1.18 to −1.06)	325,842 (229,154–437,166)	50 (35–68)	−1.12 (−1.19 to −1.06)
Male	791,359 (636,811–990,278)	382 (304–483)	26.44%	−1.28 (−1.34 to −1.22)	1,484,438 (1,407,857–1,567,816)	488 (461–516)	41.24%	−1.36 (−1.43 to −1.3)	196,945 (137,976–264,898)	65 (46–89)	−1.35 (−1.42 to −1.29)
Female	532,494 (388,981–700,577)	216 (161–284)	30.17%	−0.56 (−0.63 to −0.49)	1,007,114 (938,406–1,080,553)	268 (249–288)	38.18%	−0.71 (−0.77 to −0.64)	128,897 (91,714–172,097)	35 (25–46)	−0.71 (−0.78 to −0.65)
Western Sub-Saharan Africa											
Both	687,797 (612,198–774,831)	162 (144–181)	51.97%	−0.28 (−0.38 to −0.19)	791,303 (756,628–834,310)	256 (245–269)	80.62%	−0.11 (−0.17 to −0.06)	118,108 (82,770–159,110)	38 (27–51)	−0.1 (−0.15 to −0.05)
Male	458,218 (413,266–512,230)	220 (199–243)	54.56%	−0.18 (−0.29 to −0.07)	545,055 (517,566–576,789)	365 (348–384)	82.34%	0.09 (0.05 to 0.13)	82,176 (57,519–110,805)	54 (38–73)	0.11 (0.07 to 0.15)
Female	229,579 (197,740–263,378)	109 (92–126)	46.80%	−0.32 (−0.38 to −0.26)	246,248 (235,945–257,742)	157 (150–166)	76.82%	−0.2 (−0.24 to −0.15)	35,933 (25,140–48,506)	23 (16–31)	−0.19 (−0.23 to −0.14)

### 3.2 National-level data

**Figure 1:**
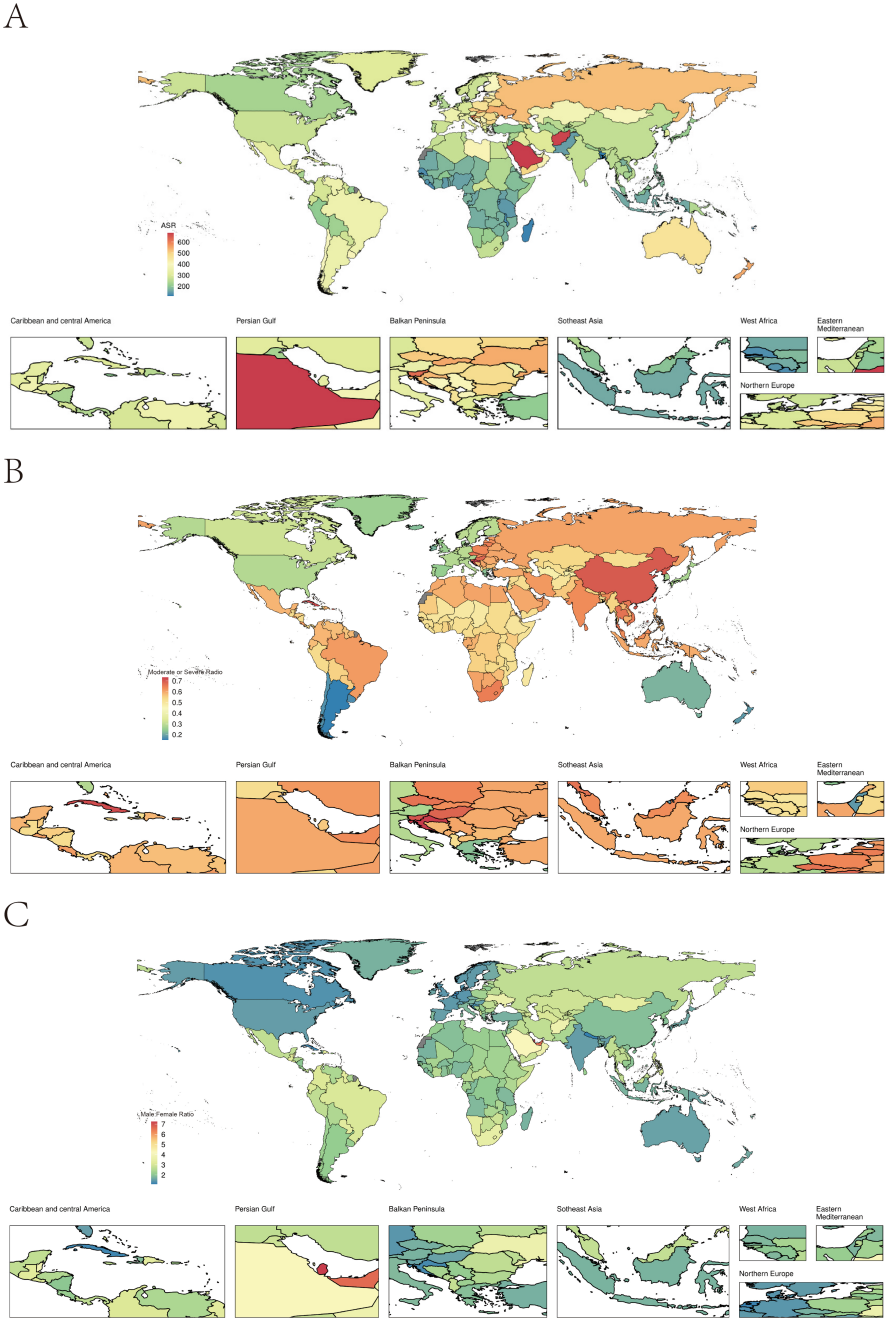
**(A)** Age-standardised incidence rates per 100,000 persons of TBI by countries/territories, 2021; **(B)** Ratio of moderate/severe TBI incidence by countries/territories, 2021; **(C)** Male to female ratio of TBI incidence by countries/territories, 2021.

Significant variations in incidence rates were observed across countries. The top three countries with the highest incidence rates (all exceeding 600 cases per 100,000 people) were Saudi Arabia (681, 95% UI: 584–777), Afghanistan (673, 95% UI: 506–941), and Slovenia (622, 95% UI: 512–736). Countries with relatively low incidence rates were predominantly located in Africa and East Asia (Figure 1A).

When examining the proportion of moderate/severe TBI in various countries, it was found that, apart from a few countries in high-income regions such as North America, Western Europe, and Oceania, the majority of countries reported moderate/severe TBI making up more than 50% of cases. Notably, in East Asian and African countries, the proportion of moderate/severe TBI was higher than expected, particularly in China, where 69.82% of TBI cases were moderate/severe. Exceptions in East Asia were observed in Japan (27.79%) and South Korea (27.20%), where minor TBI was more prevalent (Figure 1B).

Across all countries, the incidence of TBI in males was higher than in females, with more than 60% of male incidence rates being more than twice as high as those in females. Qatar and the United Arab Emirates exhibited an astonishing male-to-female incidence ratio greater than 6, far exceeding other countries (Figure 1C).

### 3.3 Age and gender perspectives

**Figure 2:**
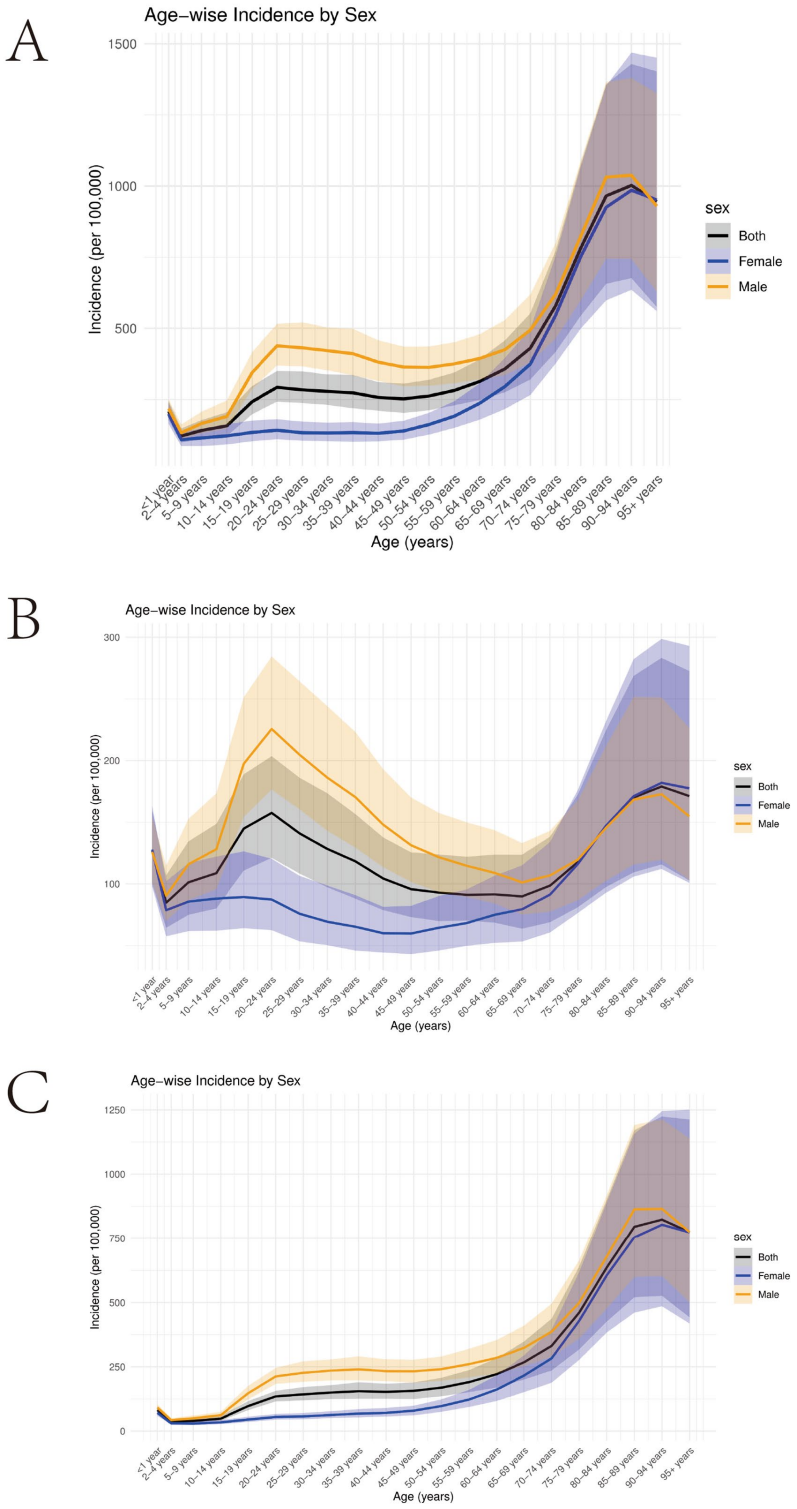
**(A)** Global incidence of TBI by age and sex, 2021; **(B)** Global incidence of minor TBI by age and sex, 2021; **(C)** Global incidence of moderate/severe TBI by age and sex, 2021.

In 2021, except for the infant population under 1 year (where head injury incidence was slightly higher due to their fragile physical condition), the incidence of head injuries increased progressively with age. The incidence was significantly higher in middle-aged and older adult individuals (40–44 years and older), reaching its peak in those aged 85 years and above. Regarding gender, male incidence rates consistently exceeded female rates. This gender disparity became significant from the 10–14 age group and began to narrow at the 70–74 age group. Notably, a small peak in incidence was observed in males aged 20–24 years, suggesting potential age-specific risk factors (Figure 2A).

Further analysis of the age distribution trends for minor and moderate/severe TBI revealed that, for minor TBI, the peak incidence in males aged 20–24 was more pronounced, with a similar trend observed in females, though the peak was lower. After this peak, the incidence of minor TBI declined until it began to rise again at ages 60–64 years for males and 45–49 years for females. For moderate/severe TBI, the incidence steadily increased with age, peaking at 90–94 years for both males and females (Figures 2B,C).

The study also analyzed the YLD trends for different severities of TBI across age groups and genders in 2021. The YLDs for both males and females increased with age, with males consistently having higher YLDs than females. This trend was particularly prominent in minor TBI, while in moderate/severe TBI, males showed the highest YLDs at 65–69 years, and females at 85–89 years, with a slight decrease thereafter (Supplementary Figure S1).

### 3.4 Injury causes

**Figure 3:**
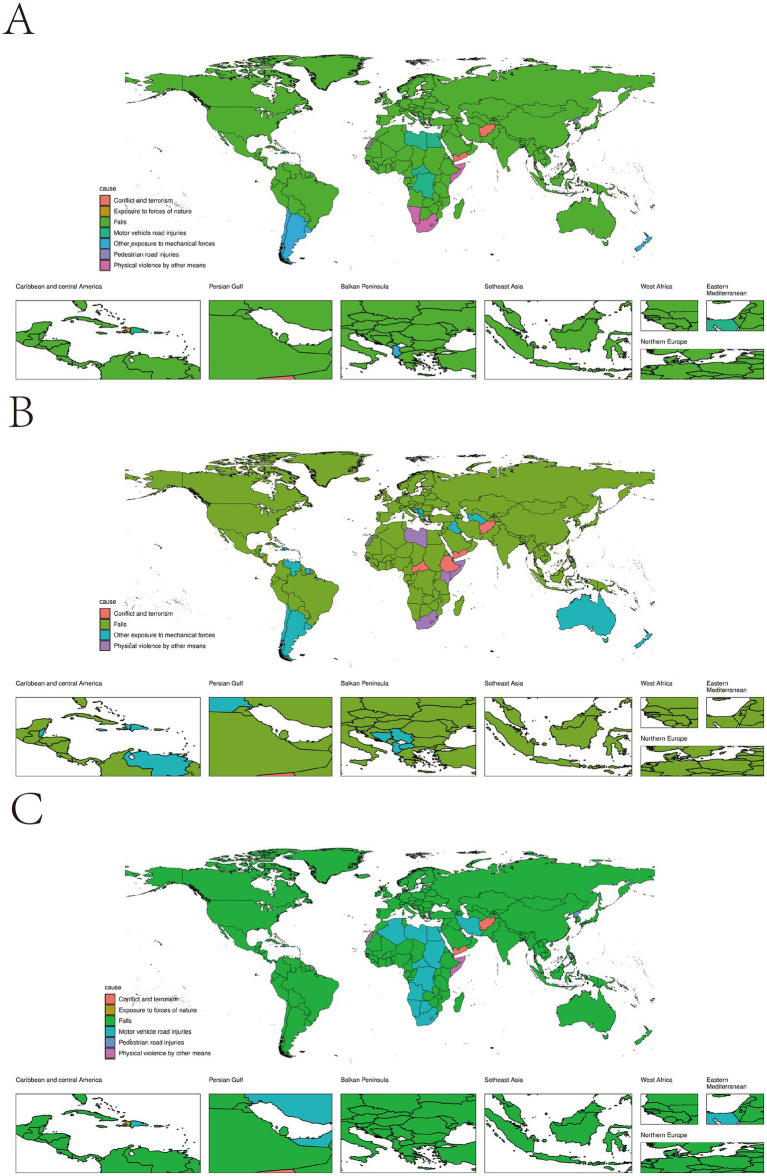
**(A)** The leading causes for age-standardised incidence rates of TBI by countries/territories, 2021; **(B)** The leading causes for age-standardised incidence rates of minor TBI by countries/territories, 2021; **(C)** The leading causes for age-standardised incidence rates of moderate/severe TBI by countries/territories, 2021.

At the national level, the primary causes of TBI across different severities were explored. The majority of countries reported falls as the leading cause, with smaller proportions attributed to traffic accidents, mechanical forces, and physical violence. Besides these, in Haiti, exposure to forces of nature was a major cause, while in Afghanistan and Yemen, conflict and terrorism were significant contributors (Figure 3A).

When comparing minor and moderate/severe TBI, falls remained the predominant cause for both types of TBI. The difference lies in that, in some African countries, moderate/severe TBI was more often caused by motor vehicle road injuries and in Central Africa and Ethiopia, most minor TBIs were caused by conflict and terrorism (Figures 3B,C).

**Figure 4:**
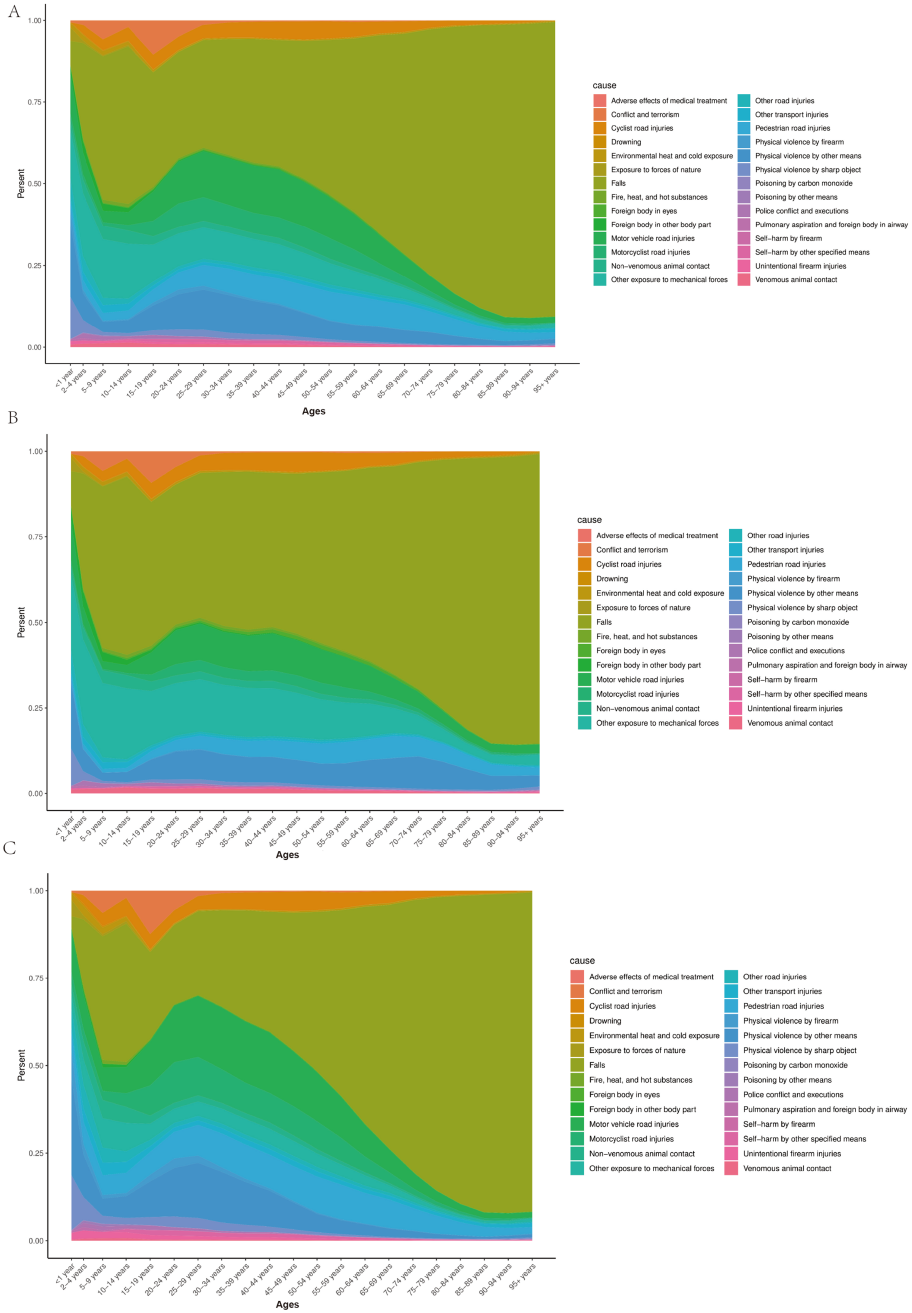
**(A)** Percentage of TBI causes for both sexes by age, 2021; **(B)** Percentage of minor TBI causes for both sexes by age, 2021; **(C)** Percentage of moderate/severe TBI causes for both sexes by age, 2021.

An analysis of the causes of TBI across different age groups revealed significant differences in the distribution of injuries. For example, traffic-related injuries (e.g., motor vehicle, bicycle, or motorcycle accidents) were major causes of head injuries in adolescents and young adults (15–29 years), while falls accounted for a larger proportion of injuries in older adults (65 years and above). For infants (<1 year), head injuries were more often caused by environmental exposures, such as falls or physical impacts. Self-harm and violence-related head injuries were more common in young and middle-aged adults (20–39 years), while head injuries due to natural forces or mechanical forces were less frequent and more evenly distributed across age groups (Figure 4A).

Further analysis of the causes of minor and moderate/severe TBI by age group revealed that the causes of minor TBI were more diverse, whereas moderate/severe TBI was more closely associated with high-intensity external forces. Traffic-related injuries were a common cause of both minor and moderate/severe TBI in adolescents and young adults, but they contributed more to minor TBI. Violent events (e.g., firearm or sharp object injuries) contributed to both minor and moderate/severe TBI in young adults (20–39 years), with a higher proportion in moderate/severe TBI cases. Falls were the primary cause of both minor and moderate/severe TBI in the older adult, with the proportion of falls leading to moderate/severe TBI significantly increasing with age (Figures 4B,C).

**Figure 5:**
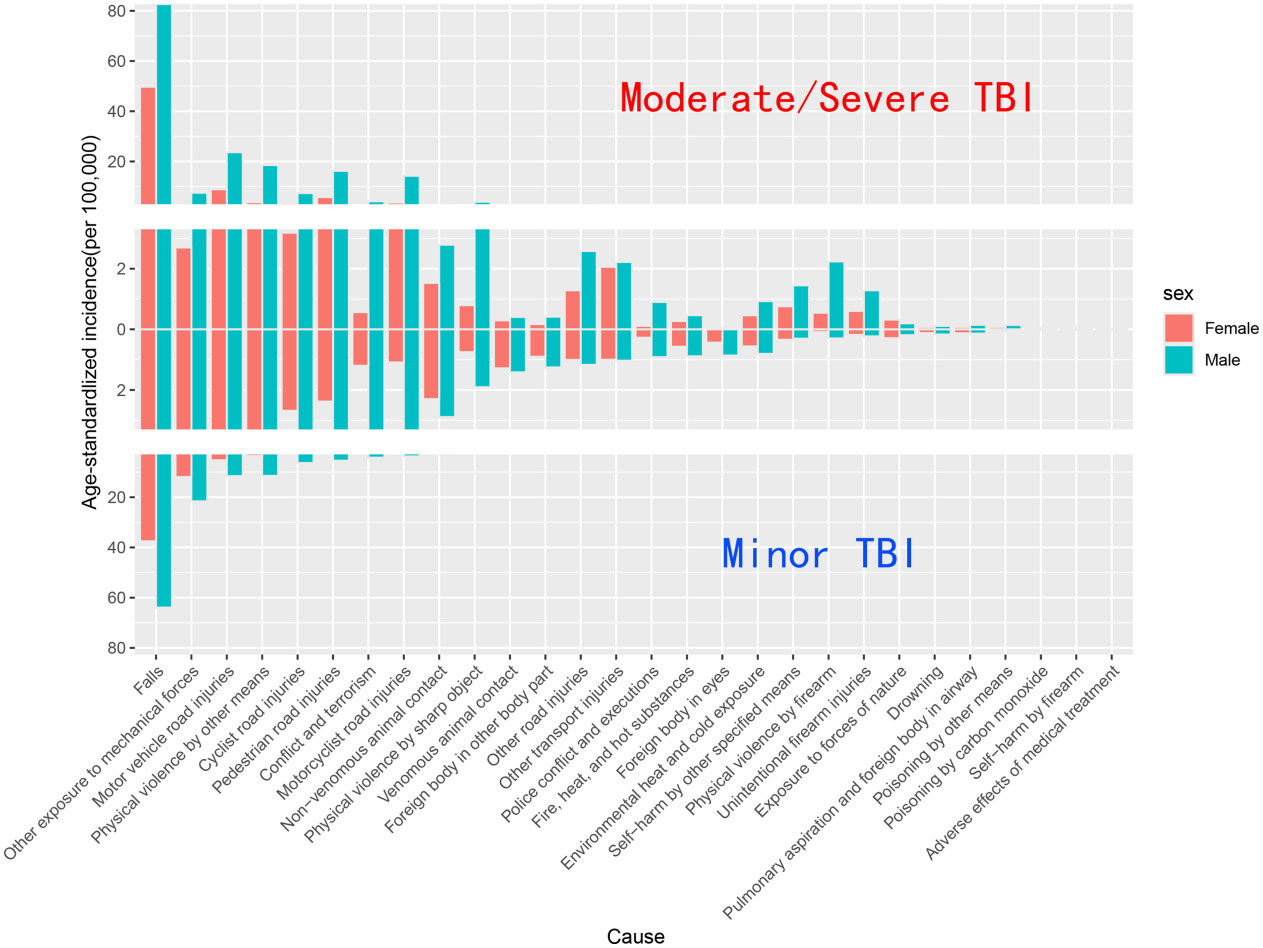
Composition of age-standardized incidence of minor and moderate/severe TBI by different causes, 2021.

When comparing male and female incidence rates for different causes of TBI, it was found that males had higher incidence rates of TBI than females across all causes, whether minor or severe. This disparity was consistent across almost all injury categories, with particularly marked differences in interpersonal violence and bicycle-related road traffic injuries. Additionally, for both minor and moderate/severe TBI, males exhibited higher rates than females in almost all causes, except for exposure to mechanical forces, non-venomous animal contact, venomous animal contact, and foreign bodies in the eyes (Figure 5).

## 4 Discussion

This study analyzes the epidemiological characteristics of TBI from 1990 to 2021 using data from the GBD database, focusing on global, regional, and national trends across different age groups, genders, and injury causes. Through descriptive statistics and modeling, we reveal the disease burden differences of TBI across various dimensions and discuss potential interventions and policy implications.

Overall, the global incidence rate of TBI has decreased over the past 30 years, reflecting the effectiveness of global prevention and management interventions. However, it is noteworthy that the proportion of moderate/severe TBI has significantly increased, suggesting that although the frequency of accidents has declined, the consequences of these incidents have become more severe. This trend varies across regions, as seen in 2021 when East Asia ranked highly in terms of TBI incidence, prevalence, and YLDs globally, which is closely linked to the region’s large population base. To better understand these differences, we need to consider multiple factors such as country, gender, age, injury cause, and the severity of TBI, thus providing a more comprehensive reflection of TBI trends.

These differences reflect the diverse TBI burden characteristics shaped by the social, cultural, and economic contexts of different countries. Due to space constraints, this article does not analyze the TBI characteristics of every country but instead focuses on three countries with the highest age-standardized incidence rates, chosen for their representativeness in various aspects.

For instance, Saudi Arabia, with the highest ASR, reports TBI mainly due to falls and traffic accidents, with traffic accidents previously being the dominant cause (15, 16). However, in recent years, with the implementation of the “Vision 2030” strategy, Saudi Arabia has responded to the WHO’s agenda for strengthening road safety through the 2030 Sustainable Development Goals. The country has significantly strengthened traffic safety regulations, and stringent traffic management measures have effectively reduced the occurrence of traffic accidents (17, 18). These measures include speed limits, mandatory seatbelt and helmet use, and a ban on drunk driving. As a result, the proportion of traffic accidents has decreased year by year, although they still rank as the second leading cause of TBI (17, 19, 20).

However, the construction process, accompanied by high-risk work environments in construction and industrial sectors, has increased the incidence of head trauma. Insufficient safety training and protective equipment may explain the rising proportion of TBI caused by falls. This suggests the need to balance national modernization with production safety, a lesson applicable to other rapidly developing countries.

In Slovenia, ranked second, the impact of geography and population structure is evident. The country is largely mountainous and located in the foothills of the Alps. The terrain and winding roads contribute to a high incidence of traffic accidents, while outdoor activities such as skiing, mountaineering, and mountain biking, popular with both locals and tourists, also carry higher accident risks. The risk of geographically-induced injury also exists in Finland, Norway, and Sweden, where cold climates and icy roads may increase the risk of falls among their populations (21).

Additionally, Slovenia’s aging population has influenced TBI incidence. In 2021, the proportion of people aged 65 and older increased from 16.37% a decade ago to 20.50% (22). Due to sarcopenia, osteoporosis, sleep disorders, comorbidities, and frailty, older adults are at higher risk of head trauma due to falls (23). This is a global issue, as the proportion of people aged 65 and above has risen from 6.2% in 1990 to 9.6% in 2021 (24). Therefore, fall prevention among older adults should be a central component of global TBI prevention strategies.

In Afghanistan, ranked third, the main cause of TBI is prolonged armed conflict (25). Other countries, such as Yemen and the Syrian Arab Republic, are similarly affected. For these countries, providing essential trauma care and rehabilitation in conflict zones remains an urgent challenge.

A comprehensive analysis of the causes, severity, and incidence of head injuries across different age groups and genders reveals that the incidence of TBI is higher in males than females in all countries worldwide. This is partly due to the differing social roles and behaviors of males and females, with traffic accidents, violence, and sports injuries more commonly associated with male-dominated activities (26).

However, in certain countries, such as Qatar and the United Arab Emirates, the gender gap is even more pronounced. This is not only due to imbalances in gender ratios but also influenced by cultural factors, with females having more limited mobility and fewer opportunities to engage in high-risk professions or activities, resulting in lower TBI incidence. Additionally, cultural influences may lead to underreporting of female injuries, particularly domestic accidents or minor TBIs. This phenomenon highlights the importance of improving the transparency and collection of TBI data, especially regarding female TBI, as a key task for future research and policy-making.

When considering age, gender differences in TBI incidence become significantly evident starting from the 10–14 age group, with males exhibiting a higher incidence rate. A small peak in incidence occurs in males aged 20–24, which is linked to their increased participation in activities such as motorcycle riding, leading to road traffic injuries. However, due to the better physical condition of younger individuals, these injuries tend to be minor TBIs. Nonetheless, improving road safety remains a critical issue. Many countries have benefited from enhanced road safety measures, such as speed limits, banning drunk driving, and requiring helmets and seatbelts, which have contributed to reducing fatal road injuries by 25%–40% (27).

However, global efforts in road safety are still insufficient. Most road infrastructure does not meet safety standards, and around 80% of roads do not meet the minimum safety requirements for pedestrians and cyclists. Legal regulations are crucial for preventing traffic fatalities, with approximately 10% of fatal traffic accidents involving drunk driving, and 50% of drivers admitting to speeding. Key legislative actions recommended by the World Health Organization include setting urban speed limits, enforcing a ban on drunk driving, and mandating helmet and seatbelt use. However, only a few countries have adopted legislative measures that align with best practices (28).

As people age, the incidence of moderate/severe TBI gradually increases, and the gender gap narrows. Older adults, due to falls, now represent the primary cause of TBI in this population. Therefore, mitigating fall-related injuries among the older adult has become a top priority in global TBI prevention. In response, the World Health Organization developed the “Falls Prevention and Management Guidelines for Older Adults” in 2022, providing risk assessment tools and intervention recommendations in areas such as lifestyle, exercise, and diet (29). At the same time, with technological advancements, many wearable devices have been introduced to the market, which have been shown to effectively detect and prevent falls (30–32). Additionally, interventions targeting cardiovascular, vestibular, and visual disorders in older adults are also believed to help prevent falls (33–35).

Overall, TBI remains a significant global safety burden. Despite the age differences in TBI cases, the mortality and disability rates associated with TBI are high, especially among the older adult. In addition to deaths caused by the injury itself within a few hours, late-stage mortality due to sepsis and multiple organ failure is also an important concern (36). Beyond epidemiological interventions and prevention, investigating and managing the injury patterns in TBI patients is also crucial. According to the latest guidelines, TBI patients should undergo timely evaluation, monitoring of vital signs and Glasgow Coma Scale scores, and rapid triage and transport (37, 38). For patients exhibiting altered mental states, loss of consciousness, post-traumatic amnesia, or focal neurological deficits, a head CT scan is required (39). Monitoring blood pressure and intracranial pressure is necessary to ensure adequate cerebral perfusion (40). Surgical removal of large traumatic hematomas should be performed before neurological deterioration occurs (41). In addition to specialized treatments, life support, airway management, and nutritional supplementation are also indispensable components of TBI patient care.

Furthermore, with the development of bioinformatics-driven research, biomarkers have emerged as important indicators for assessing brain injury. Advances in forensic pathology have highlighted the applications of tissue pathology and immunohistochemical biomarkers in improving TBI diagnosis and prognosis. Identifying specific biomarkers can help determine the severity of injury and post-traumatic survival, ultimately contributing to better patient outcomes and cost-effective healthcare strategies (36). Widely accepted brain injury-related biomarkers include glial fibrillary acidic protein (GFAP), ubiquitin carboxyl-terminal hydrolase L1 (UCH-L1), and S100 calcium-binding protein (S100B) (42–46). Notably, recent studies have also revealed the role of circulating microRNAs (miRNAs) in TBI pathophysiology. Specific miRNAs, such as miR-34b, miR-34c, miR-135a, miR-200c, and miR-451a, exhibit dynamic expression changes after injury, which may promote the development of new therapeutic strategies for TBI management (47).

Despite providing comprehensive epidemiological data on TBI, this study has several limitations. First, the GBD 2021 database did not link TBI with behavioral risk factors such as alcohol consumption, making it impossible to assess the specific impact of these factors on TBI incidence. For example, alcohol significantly increases the risk of TBI, but due to the lack of relevant data, we could not analyze its role in TBI occurrence. Second, the GBD data largely depend on reports from healthcare institutions, and some TBI patients, especially those with minor injuries who did not seek treatment, are not recorded. As discussed earlier, the underreporting rate in some countries is even strongly associated with gender, which affects the accuracy of the final statistics. Third, GBD data categorize TBI into Minor TBI and Moderate/Severe TBI, but do not further separate Moderate from Severe, making the differences between them unnoticeable. Last, the data, which ends in 2021, is not recent, so it does not fully reflect the current epidemic distribution of TBI.

## 5 Conclusion

In summary, this study leverages the latest GBD data from 2021 to provide a comprehensive analysis of different severity of TBI across different countries, age groups, and genders. The investigation into the distribution and variation tendency of TBI offers novel insights into the global burden of this injury. By examining the underlying causes of TBI across diverse populations, this manuscript not only enhances our understanding of TBI patterns but also identifies key factors contributing to its prevalence. The findings underscore the urgent need for tailored prevention strategies that address the specific risk factors in various demographics. Furthermore, the study’s results provide crucial evidence for policymakers and healthcare providers to design more effective intervention strategies aimed at reducing the incidence and societal burden of TBI. This work significantly contributes to the existing literature by offering an updated, detailed analysis of global TBI trends, which can guide future research and improve long-term public health strategies.

## Data Availability

The original contributions presented in the study are included in the article/Supplementary material, further inquiries can be directed to the corresponding author.

## Footnotes

^1^ http://ghdx.healthdata.org/gbd-results-tool

## Supplementary material

SUPPLEMENTARY FIGURE S1 **(A)** Global YLDs of TBI by age and sex, 2021; **(B)** Global YLDs of minor TBI by age and sex, 2021; **(C)** Global YLDs of moderate/severe TBI by age and sex, 2021.
