# Supplementary figures and images for: Global, regional, and national burdens of traumatic brain injury from 1990 to 2021
Source: Front Public Health. 2025 Apr 14;13:1556147. doi: 10.3389/fpubh.2025.1556147 (PMC12034675; doi:10.3389/fpubh.2025.1556147)

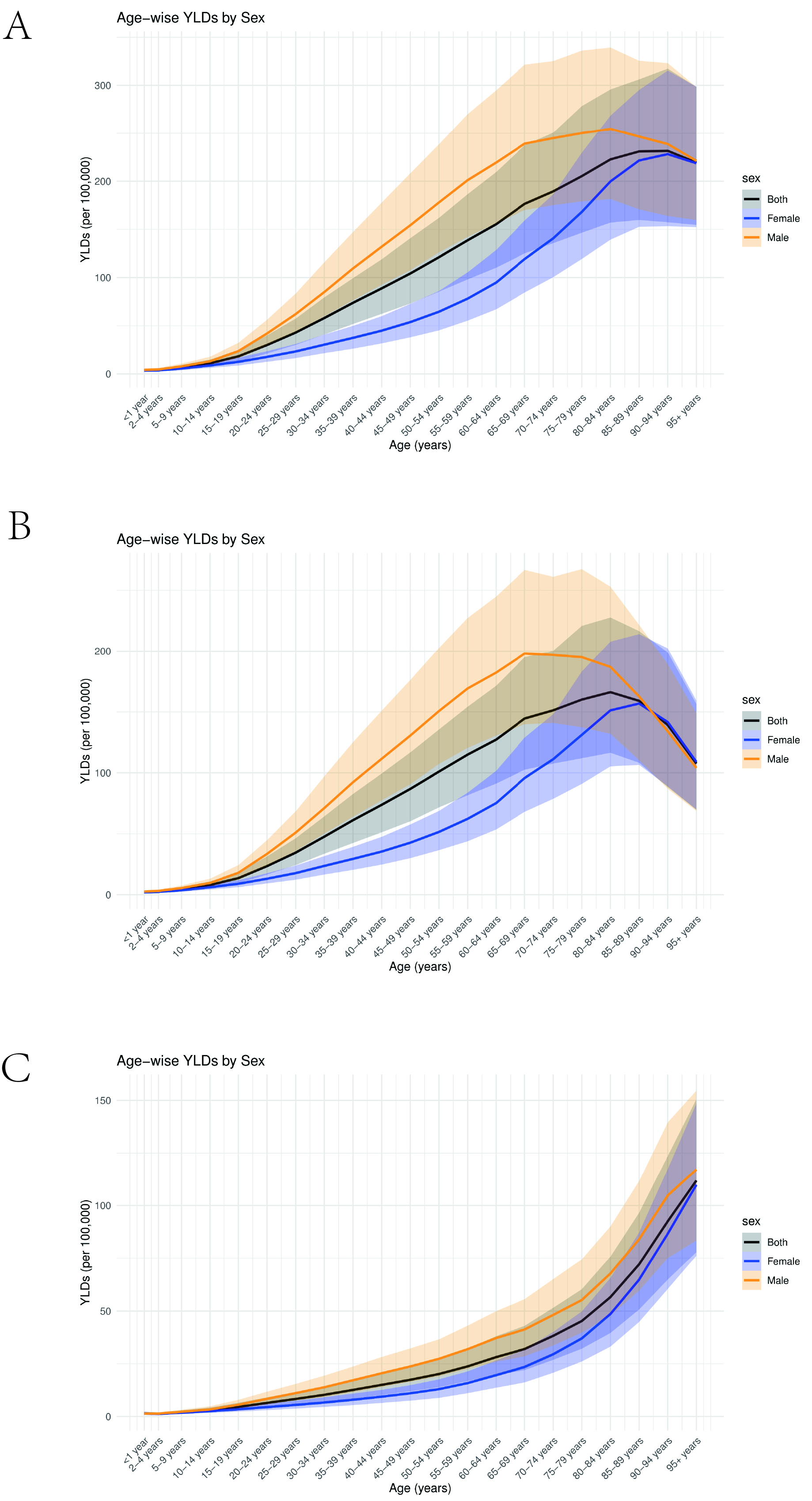

Supplement: SUPPLEMENTARY FIGURE S1 — (A) Global YLDs of TBI by age and sex, 2021; (B) Global YLDs of minor TBI by age and sex, 2021; (C) Global YLDs of moderate/severe TBI by age and sex, 2021. [file Image_1.tif]
